# An experimental study on strength improvement of expansive subgrades by polypropylene fibers and geogrid reinforcement

**DOI:** 10.1038/s41598-022-10773-0

**Published:** 2022-04-23

**Authors:** Nitin Tiwari, Neelima Satyam

**Affiliations:** Department of Civil Engineering, Indian Institute of Technology Indore, Indore, India

**Keywords:** Engineering, Civil engineering

## Abstract

The rapid development of infrastructure often encounters the loose subgrades and is becoming difficult to carry to construction activities. Numerous counteracting methods are developed to control the swelling-shrinkage behavior of the expansive subgrades. The mechanical stabilization of the expansive subgrades by reinforcing with the polypropylene fiber and geogrid is sustainable. Geogrids and polypropylene fibers have been used extensively to strengthen the expansive subgrade and foundations as individuals. The polypropylene fiber reinforcement enhanced the reinforced expansive subgrades's tensile strength capacity, wherein the geogrid reinforcement is the quick fix mechanical stabilization technique, which reduces the pavement failures. In this research, the polypropylene fiber and geogrid reinforcement’s combined effect has been evaluated to stabilize the pavement subgrades. The various mechanical strength test such as unconfined compressive strength (UCS) and large direct shear box test was conducted to evaluate the mechanical interaction between expansive subgrades, polypropylene fiber, triaxial geogrid, and biaxial geogrid at the interface. The polypropylene fiber of 12 mm length was used in the proportion of 0.25%, 0.5%, and 1.0% and single geogrid layer at mid-depth. The result shows that reinforced subgrades’ shear strength with a layer of biaxial/ triaxial geogrid and polypropylene fiber increases by 177%. It is also observed that the unconfined compressive strength of the expansive subgrades increased ranging 3.8–139.6% with the inclusion of polypropylene fiber with geogrid in different combinations. The combined reinforcement method shows an effective treatment methodology to improve the property of expansive subgrades.

## Introduction

Expansive subgrades are characterized by the nature of changing their volume with the change in water content. This volume change is known as the swelling-shrinkage behavior of expansive subgrades, and hence expansive subgrades are also known as swell-shrink subgrades^1–4^. Pavement distresses in roadways are one of the impacts of expansive soil in the subgrades layer^5–7^. In a dry state, expansive subgrades initiate shrinkage cracking, which propagates through the pavement system and leads to longitudinal, transverse, and fatigue cracking and rutting in the case of pavement surface^8^. Structures also suffer comparatively extensive damage when constructed on highly plastic clay subgrades, as such subgrades undergo cycles of wetting and drying. Therefore, such characteristics of expansive fine-grained subgrades are one of the most significant reasons which lead to cracks, distress, and most of the damage^9^.

Expansive subgrades lie mostly in the central and the western part and cover more than 15–20% of India's geographical area^10,11^. Expansive subgrades are also known as weak subgrades because upward swelling pressure increases the expansive subgrades' moisture content, causing failure. The expansive subgrades engineering properties are improved using several reinforcing methods. Numerous counteracting methods are developed to control the swelling-shrinkage behavior of the expansive subgrades^12–19^. Geogrid reinforcement is another widely used method to improve the engineering properties of weak subgrades^20–23^. The geogrid improves the engineering properties of the foundation by mechanical stabilization^24–27^. Several studies have been carried out to understand the geosynthetic reinforced expansive subgrades behavior^21,28–32^. The use of geogrid improves the service life of the pavement structure by reducing the reflective crack and reinforcing the subgrade materials^33–38^. The study carried out by Jahandari et al.^39^ presented the long-term performance of the geogrid reinforced expansive subgrades and showed sustainable use of the geogrid to reinforced the lime treated expansive subgrades. Similarly, Chenari et al.^40^ studied the combined effect of geogrid and expended polystyrenes (EPS) to improve the cyclic interface behavior of reinforced expansive subgrades. The long-term performance analysis of geosynthetic reinforced expansive subgrades has been studied by Roodi and Zornberg^24^ and shows the method has cost-effective and sustainable.

The fiber reinforcement of the expansive subgrades has proven to be another inexpensive and suitable method. Various researchers carried out extensive studies to investigate the effectiveness of the fiber in improving the lifespan of the expansive subgrades^41–46^. The polypropylene fiber made up of waste plastic has been effectively used as reinforcement^47–51^. Tiwari et al.^52^ investigated the effect of polypropylene fibers on the reinforcement of the expansive subgrades stabilized with silica fume and found a significant improvement in the engineering properties under freeze-thaw cycles. Deng et al.^53^ studied the effect of polypropylene fiber to reinforced the expansive subgrades and found that the strength has been increased with the increase of fiber length at optimum moisture content.

It has been observed that various researchers have studied the combined effect of different materials with the geogrid to achieve sustainability. However, the combined effect of polypropylene fiber and geogrid has not been well studied. The interface behavior and shear strength properties of polypropylene fiber and geogrid-reinforced expansive subgrades are poorly explored. Therefore, to study the combined reinforcement of fibers and geogrids, a detailed experimental study was conducted in this research. The polypropylene fiber and the geogrid effect were studied by carrying out unconfined compressive strength and large direct shear tests. Shear strength was assessed by placing the biaxial and triaxial geogrid in the center and reinforcing the expansive subgrades with 0.25%, 0.50% and 1.0% polypropylene fiber.

## Materials properties

### Expansive subgrades

The subgrades materials used in this research have been collected from Madhya Pradesh (India). The collected expansive subgrades' index properties have been investigated, and it has been observed that the subgrades is classified as high plasticity clay (CH) as per unified soil classification system (USCS). The grain size distribution of the obtained expansive subgrades shows the presence of clay (71.5%), silt (24.5%), and sand (4.0%). The presence of higher clay content exhibits a higher swelling pressure; therefore, the free swell index of 120% has been observed. The index properties of the subgrades considered in the study has been shown in Table 1.

**Table 1:** Index properties of expansive subgrades considered^11^.

Property	Value
Specific gravity	2.78
Liquid limit (%)	89
Plastic limit (%)	47
Plasticity index (%)	42
Shrinkage limit (%)	11
USCS classification	CH
**Grain size distribution**	
Clay (%)	71.5
Silt (%)	24.5
Sand (%)	4.0
Free swell index (%)	120

### Polypropylene fiber

 12 mm long Polypropylene fibers made up of waste plastic have been considered in this research. Fiber with a melting point of 165 °C and with 910 kg/m^3^ bulk density is used. PP fiber having higher tensile strength and are non-corrosive. Various properties of the PP fiber considered in this study are shown in Table 2.

**Table 2:** Properties of polypropylene fiber considered^45^.

S. no	Property	Value
1	Specific gravity	0.91
2	Tensile strength (kN/mm^2^)	0.67
3	Young’s modulus (kN/mm^2^)	4.0
4	Melting point (°C)	165
5	Ignition point (°C)	600
6	Bulk density (kg/m^3^)	910
7	Loose density (kg/m^3^)	250–430
8	Fiber cut length (mm)	6 mm
9	Dispersion	Excellent
10	Acid and salt resistance	Chemical proof

### Geogrid

**Figure 1:**
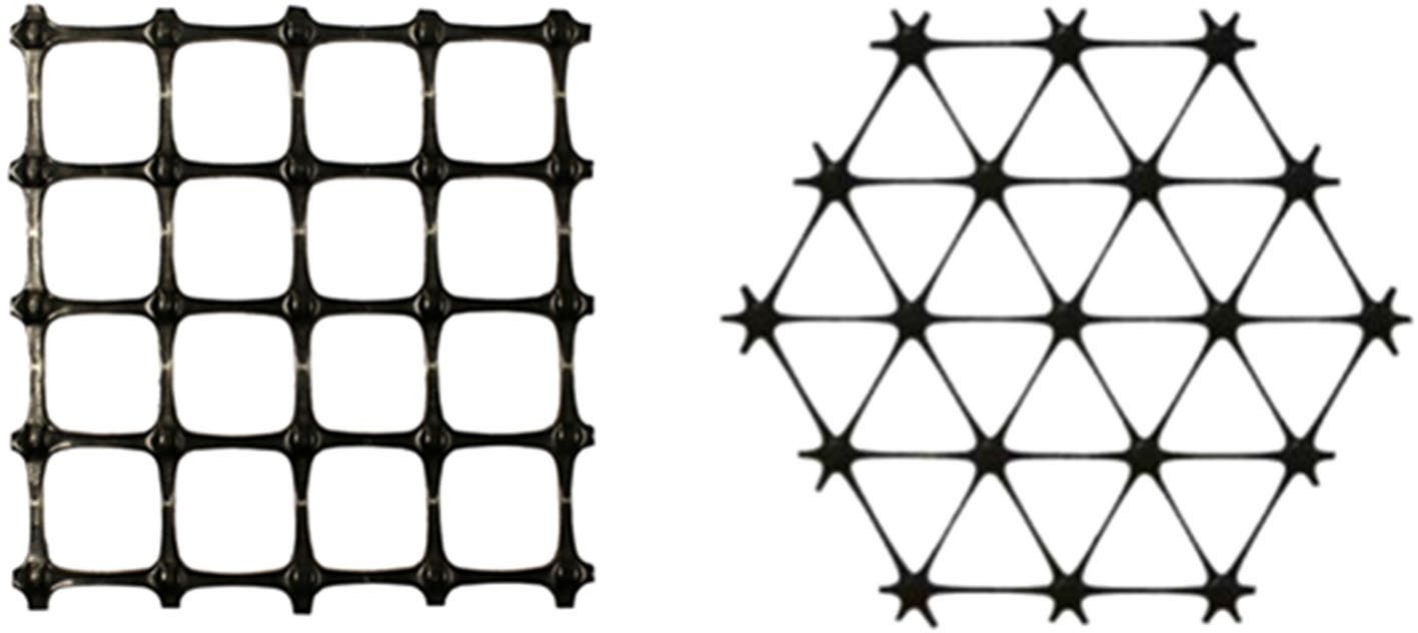
Biaxial and triaxial geogrid.

Biaxial and Triaxial polypropylene geogrid that is integrally formed by punch and drawn and extrusion process as shown in Fig. 1 has been used in the study. Index properties of biaxial and triaxial geogrid are presented in Tables 3 and 4 as informed by Tensar Geosynthetics India Pvt. Ltd.

**Table 3:** Properties of biaxial geogrid considered.

Characteristics			Units	MD	CD
Mechanical properties	Tensile strength ASTM 6626	@ 2% Strain	kN/m	4.1	6.6
		@ 5% Strain	kN/m	8.5	13.4
		Ultimate	kN/m	12.4	19
	Junction efficiency		%	93	-
	Flexural stiffness		mg-cm	250,000	-
Geometric properties	Aperture dimensions		mm	25	33
	Minimum rib thickness		mm	0.76	0.76
	Rib width		mm	3.2	3.2
Polymer type			Polypropylene		
Manufacturing process			Integrally formed biaxial geogrid

**Table 4:** Properties of triaxial geogrid considered.

Characteristics			Unit	Value
Geometric properties	Rib pitch	Longitudinal	mm	40
		Diagonal	mm	40
	Mid-rib depth	Diagonal	mm	1.6
		Transverse	mm	1.4
	Mid-rib width	Diagonal	mm	1
		Transverse	mm	1.2
	Aperture shape		–	Triangular
Structural integrity	Junction efficiency		%	93
	Radial stiffness @ 0.5% strain		kN/m	300
Polymer type			Polypropylene	
Manufacturing process			Integrally formed Triaxial geogrid	

## Experimental program

**Figure 2:**
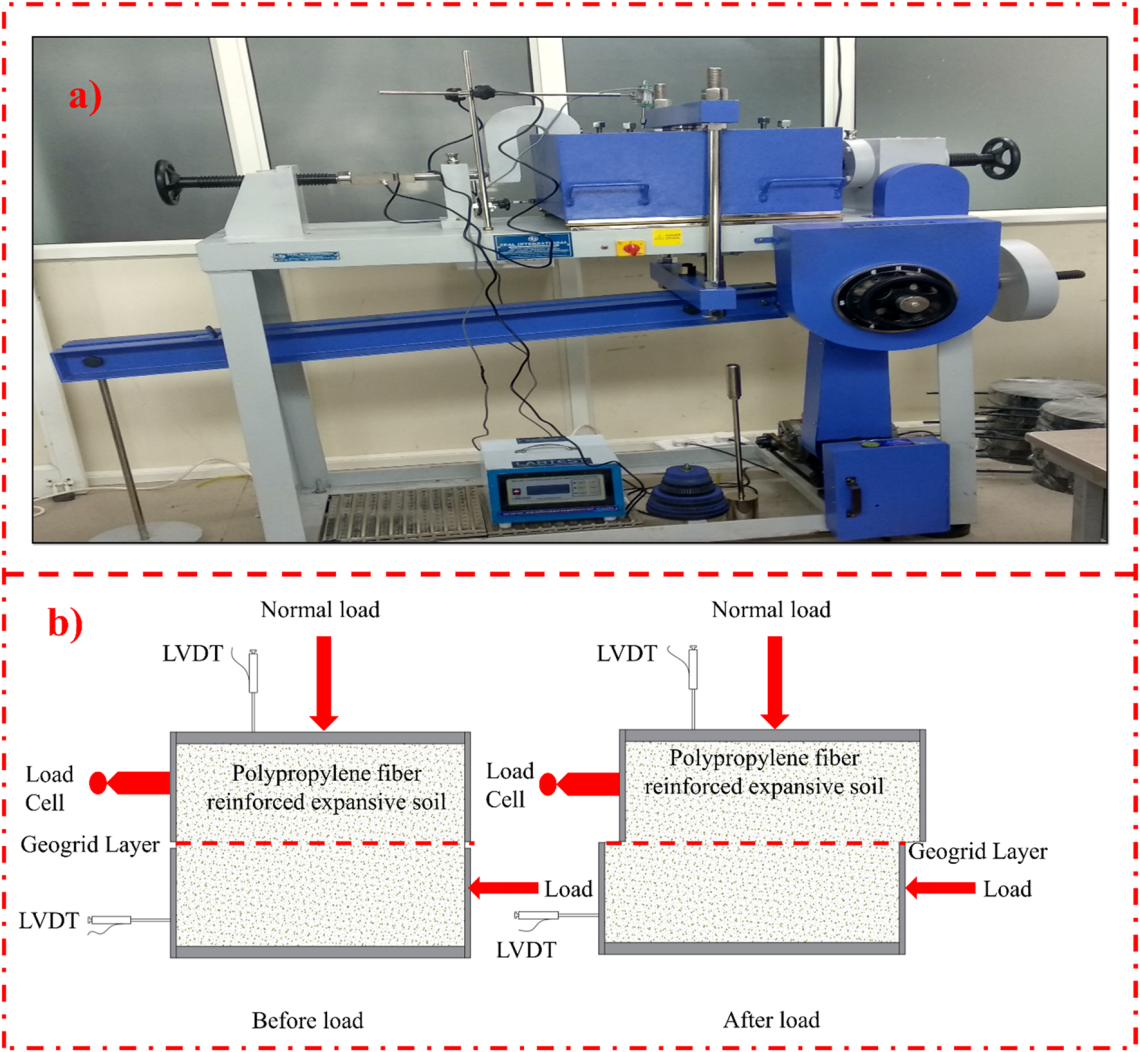
Experimental Setup for the large direct shear test (**a**) equipment used (**b**) shear box arrangement.

In this study, the engineering properties of polypropylene fiber and geogrid reinforced expansive subgrades were investigated. Unconfined compressive strength and large size direct shear tests have been conducted to investigate the shear strength and compressive strength of the reinforced expansive subgrades specimen. The index properties i.e. optimum moisture content (OMC), maximum dry density (MDD), liquid limit (LL), plastic limit (PL), grain size distribution (GSD), specific gravity, free swell index, were investigated to characterize the expansive subgrades. In the initial phase, the expansive subgrades were mechanically reinforced with 0.25%, 0.50%, and 1.00% polypropylene fiber content. The fiber content and the fiber length has been choosen as per the detailed study proposed by Tiwari et al.^52^. The water content plays a vital role in the performance of the expansive subgrades. Various studies conducted on expansive subgrades show that the subgrades exhibit the maximum strength at the optimum moisture content. Therefore, all the specimens were prepared at the optimum moisture content to assess PP fiber and geogrid reinforcement's behavior. The PP fiber in the required quantity has been mixed in the mixture with optimum moisture content and then kept in the humidity cabinet at 27± 2 °C temperature and 65 ±5%. The obtained soil-PP fiber has been compacted in the desired shape and size using lightweight compactor. The polypropylene fiber and the geogrid effect were studied by carrying out unconfined compressive strength and large direct shear tests. Shear strength was assessed by placing the biaxial and triaxial geogrid in the center and reinforcing the expansive subgrades with 0.25%, 0.50% and 1.0% polypropylene fiber. The large direct shear box of 300 mm × 300 mm × 150 mm is used to evaluate the effect on expansive subgrades geogrid interaction behavior (Fig 2a). The lower box size was kept large than the upper shear box to maintain an equal shear area during the experiment (Fig. 2b). The 287, 335, and 383 numbers of blow were applied to achieve the dry density using the lightweight proctor method. The effect of the biaxial and triaxial geogrid has been assessed by placing the geogrid at mid-depth of the direct shear specimen. The direct shear test has been carried out at the strain rate of 1.25 mm/min as per Indian Standard and by applying the three different normal loads.

The maximum shear load and displacement during the experiment were recorded with LVDT and load cell of capacity 50 mm and 50 kN, respectively. The stress-strain behavior of the reinforced and unreinforced samples has been investigated by conducting the unconfined compressive strength test of 50 mm diameter. The samples were prepared as per the test sections mentioned in Table 5. The stress was calculated by applying the 1.25 mm/min constant strain rate up to deviator stress. The single-layer geogrid placed at the mid depth to evaluate the effect on unconfined compressive strength. In this research, the expansive subgrades, biaxial geogrid, triaxial geogrid, and polypropylene fiber are referred to as BC, BG, TG, and PP, respectively.

**Table 5:** Reinforced and unreinforced section considered.

Polypropylene fiber	Type of geogrid		
	No geogrid	Biaxial geogrid	Triaxial geogrid
0.00%	BC	BC + BG	BC + TG
0.25%	BC + 0.25% PP	BC + 0.25% PP + BG	BC + 0.25% PP + TG
0.50%	BC + 0.50%PP	BC + 0.50%PP + BG	BC + 0.50%PP + TG
1.00%	BC + 1.00%PP	BC + 1.00%PP + BG	BC + 1.00%PP + TG

## Results and discussions

**Figure 3:**
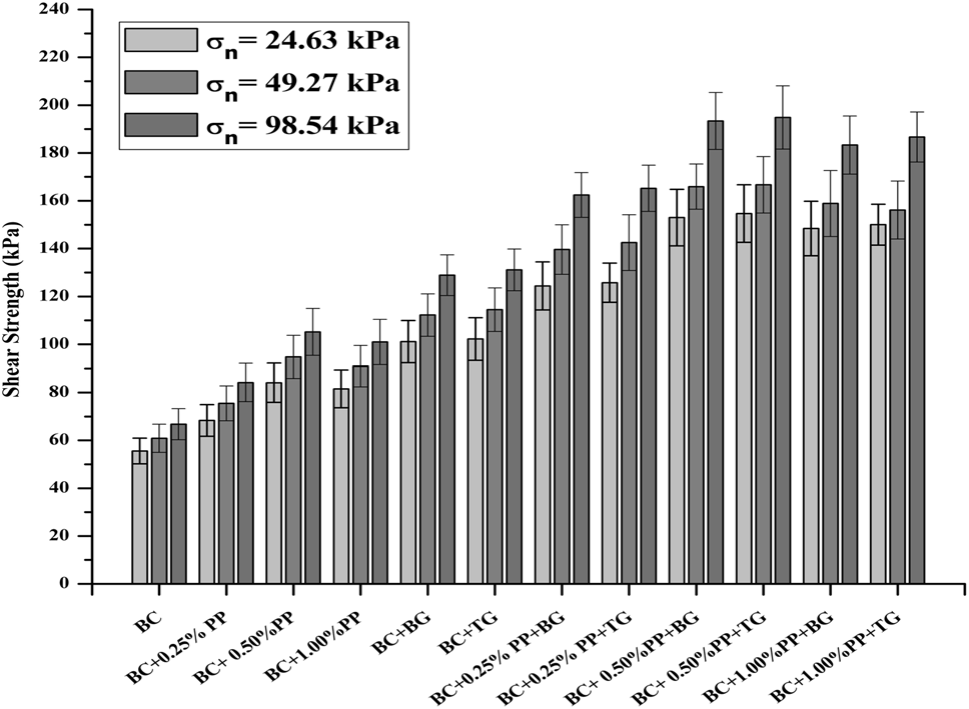
Shear strength of reinforced and unreinforced expansive subgrades.

The shear strength of reinforced and unreinforced expansive subgrades has been evaluated by direct shear and unconfined compressive strength tests. An attempt has been made to assess the stress–strain, and interfacial frictional behavior of geotextile reinforced expansive subgrades. The unconsolidated undrained direct shear test has been conducted at a strain rate of 1.25 mm/min to obtain the shear strength parameters between soil-soil, soil-fiber and soil-geogrid. The large-size direct shear test resembles the field condition of the subgrade. The frictional resistance obtained from large-size direct shear is the combined effect of the soil-soil and soil-geotextile interaction^11,54^. The shear strength of PP fiber and geogrid-reinforced and unreinforced expansive subgrades is shown in Fig. 3. The variation in fiber content was presented together with the biaxial and triaxial geogrid. The direct shear test results show that the PP fiber and geogrid effectively improve the strength of the expansive subgrades. The expansive subgrades have been reinforced by placing the biaxial and triaxial geogrid and the mid-depth. The shear strength has been improved from 55.56 to 101.11 kPa (82%), and 102.22 kPa (84%) while 68.33 kPa (23%), 84.05 kPa (51.29%), and 81.53 kPa (46.75) with the inclusion of 0.25%, 0.50%, and 1.00% polypropylene fiber at 24.63 kPa normal stress respectively. The shear strength improvement can be attributed to the frictional resistance of expansive subgrades and geogrid layers. Also, the inclusion of the PP fiber content reinforced the expansive subgrades particle and offers resistance. The dilation property plays a significant role in investigating expansive subgrades fiber reinforcement's interaction behavior^55^. The discrete distribution of the PP fiber in the expansive subgrades acted as a spatial network interlocked with the subgrades and formed a strong bond. The fiber-reinforced expansive subgrades offers relatively higher resistance against the applied force. The rearrangement of the soil particles occurs with the increasing strain rate; at this stage, the fiber's tensile strength effectively intact the soil specimen and remains intact against the applied load^56^.

The shear strength has been increased by 60 kPa, 112 kPa, 114 kPa in the case of biaxial and 66 kPa 128 kPa, 131 kPa in the case of triaxial geogrid under the normal pressure of 49.27 kPa and 98.54 kPa. Similar pattern has been observed with the inclusion of propylene fiber content (i.e. BC+0.25% PP, BC+ 0.50%PP, BC+1.00%PP), shear strength increases between 10.5 and 12.5% under 49.27 kPa and 98.54 kPa normal stress. The shear strength of reinforced expansive subgrades specimens improved due to apparent cohesion similar to the unreinforced expansive subgrades specimen. Due to high confining pressure, the apparent cohesion between soil and geotextile has been improved.

A higher tensile strength of geotextile with strong interaction with clay particles enhanced the shear strength of reinforced specimens^57^. The results also show the potential increment in the shearing strength with both geogrid and polypropylene fiber are used to reinforce the expansive subgrades. It can be noted that with the inclusion of fiber content with geogrid, the shear strength increases exponentially; however, the effect of biaxial and triaxial geogrid is similar. The interface shear strength coefficient α, has been calculated using Eq. (1) to quantify the effect of the geogrid reinforcement.

1 $$\upalpha = \frac{{\uptau_{{{\text{reinforced}}}} }}{{\uptau_{{{\text{unreinforced}}}} }}$$

where τ reinforced is the shear strength of expansive subgrades reinforced with polypropylene fiber and geogrid at the interface, and τunreinforced is the shear strength of expansive subgrades.

Table 6 summaries the measured maximum and average shear strength coefficients for normal stresses of 24.69 kPa, 49.27 kPa and 98.54 kPa. The average peak interface shear strength coefficients of the subgrades with polypropylene fiber and geogrids used in this study range from 1.24 to 2.81. The average peak interface shear strength coefficients are lowest for BC + 0.25%PP interface and highest for BC + 0.50%PP + TG interface. The higher value of the $\upalpha$ represents the maximum improvement in the reinforcement. If the value of $\upalpha$ is observed less than one, then it indicates the loss in strength. The values of $\upalpha$ with varying normal pressure have been shown in Table 6. At normal pressure 24.69 kPa, soil particles are dilative and, therefore, less shear strength observed. However, with the increase in the normal pressure, soil particles got intact and exhibited a higher strength. It can also be observed that the soil-soil and soil-fiber interlocking system also significantly influence the shear strength of the reinforced expansive subgrades. The triaxial geogrids are performed better than the biaxial geogrids, which indicates that the aperture size and type of geogrid also play a vital role in the reinforcement of the expansive subgrades. It can be concluded that based on the interlocking mechanism, the shear strength properties changed in the reinforced expansive subgrades.

**Table 6:** maximum shear strength coefficients for normal stresses of 24.69, 49.27 and 98.54 kPa.

Reinforced section	Maximum shear strength coefficient α_peak_			
	Normal stress (σ_n_) kPa			Average α_peak_
	24.69	49.27	98.54	
BC	1.00	1.00	1.00	1.00
BC + BG	1.82	1.84	1.93	1.86
BC + TG	1.84	1.88	1.96	1.89
BC + 0.25% PP	1.23	1.24	1.26	1.24
BC + 0.25% PP + BG	2.24	2.29	2.43	2.32
BC + 0.25% PP + TG	2.26	2.34	2.47	2.36
BC + 0.50%PP	1.51	1.56	1.57	1.55
BC + 0.50%PP + BG	2.75	2.72	2.90	2.79
BC + 0.50%PP + TG	2.78	2.74	2.92	2.81
BC + 1.00%PP	1.47	1.49	1.51	1.49
BC + 1.00%PP + BG	2.67	2.61	2.75	2.68
BC + 1.00%PP + TG	2.70	2.56	2.80	2.69

**Figure 4:**
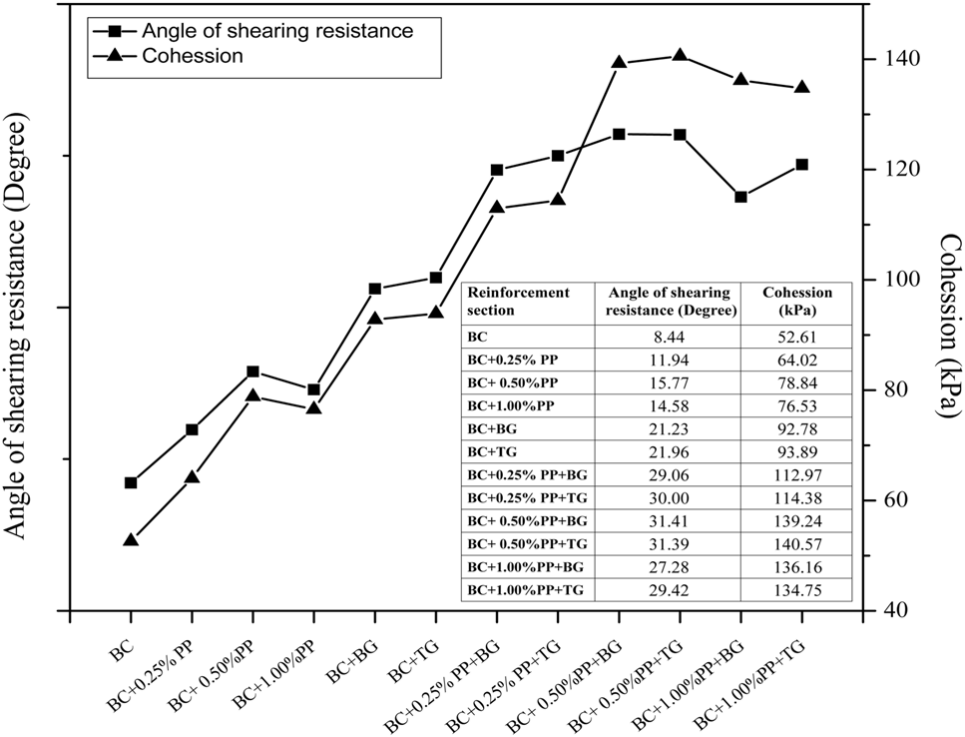
Cohesion and angle of shearing resistance of reinforced and unreinforced expansive subgrades.

Figure 4 shows the cohesion and angle of shearing resistance of reinforced and unreinforced expansive subgrades. The improvement in the angle of shearing resistance and cohesion has been observed. The shear resistance angle was improved for the biaxial geogrid from 8.44° to 21.23° and for the triaxial geogrid from 8.44° to 21.96°. Similarly, when the expansive subgrades were reinforced with 0.50% PP fibers, the shear resistance angle increased from 8.44° to 31.49° in the case of the biaxial geogrid and from 52.61 to 92.78 kPa in the case of the triaxial geogrid. At the same time, cohesion force was improved for the biaxial geogrid from 52.61 to 93.89 kPa and for the triaxial geogrid from 8.44° to 21.96°. Similarly, when the expansive subgrades were reinforced with 0.50% PP fibers, the shear resistance angle increased from 52.61 to 139.24 kPa in the case of the biaxial geogrid and from 52.61 to 140.57 kPa in the case of the triaxial geogrid.

The improvement in the angle of shear resistance and cohesion force can be attributed to the interlocking pattern of the soil-soil, soil-fiber, and soil-geogrid. The interface efficiency for the reinforced section observed in a large direct shear test is higher than the results reported by Abu-Farsakh et al.^58^. The results ascertained the effective utilization of the geogrid layer to improve the shear strength of the expansive subgrades.

**Figure 5:**
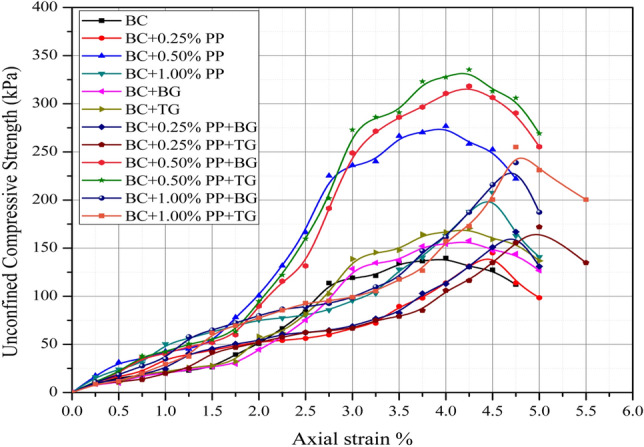
Unconfined compressive strength of reinforced and unreinforced expansive subgrades.

The unconfined compressive strength (UCS) curve of reinforced and unreinforced expansive subgrades is shown in Fig. 5. Significant strength improvement has been observed with the inclusion of polypropylene fiber and geogrid. The UCS value of the unreinforced section was observed as 139.7624 kPa, which increase ranging 145.15–335 kPa.

The maximum value of reinforced section BC+ 0.50%PP+TG can be considered the optimum percentage for reinforcement. The geogrid effect does not affect much the material’s strength; however, the layer creates a strong base and separates the section into two parts. With the inclusion of the geogrid layer, the L/D ratio of layers changes, and as a result, the increment in the UCS value is observed. The UCS of the polypropylene fiber mix increased with the increment of the PP fiber content; however, at a higher amount of PP fiber content, the axial strain capacity of the expansive subgrades reduces.

## Conclusions

The expansive subgrades pose distress on the pavement structure and causes failure due to the swelling shrinkage nature. This study investigated the coupling effect of the polypropylene fiber and geogrid reinforcement on expansive subgrades. The shear strength of the reinforced expansive subgrades has been assessed by large direct shear strength and unconfined compressive strength tests. The combined use of the PP fiber and geogrid ascertained a significant improvement in the expansive subgrades engineering properties. The use of biaxial and triaxial geogrid significantly improves the angle of shearing resistance of PP fiber reinforced and unreinforced expansive subgrades. The improvement has been attributed to the interlocking mechanism among soi-soil, soil-fiber and soil-geogrid. The shear strength of the expansive subgrades with the inclusion of PP fiber and geogrid increase up-to 154 kPa from 55.43 kPa. The 0.50% PP fiber gives optimum results with biaxial and triaxial geogrid. However, the biaxial and triaxial geogrid performed; similarly, no significant improvement has been observed. The unconfined compressive strength of the reinforced section increases with the addition of geogrid and PP fiber. The UCS value of the unreinforced section was observed as 139.7624 kPa, which increase ranging 145.15–335 kPa.
